# Malignant Rheumatic Heart Disease Presenting as Quadrivalvular Stenosis

**DOI:** 10.14740/cr437w

**Published:** 2015-12-16

**Authors:** Santosh Kumar Sinha, Ramesh Thakur, Vinay Krishna, Chandra Mohan Varma, Amit Goel, Mukesh Jitendra Jha, Ashutosh Kumar, Vikas Mishra, Pradyot Tiwari, Rupesh Sinha

**Affiliations:** a Department of Cardiology, LPS Institute of Cardiology, G.S.V.M. Medical College, Kanpur, Uttar Pradesh 208002, India; b LPS Institute of Cardiology, G.S.V.M. Medical College, Kanpur, Uttar Pradesh 208002, India

**Keywords:** Quadrivalvular stenosis, Rheumatic heart disease, Tricuspid stenosis, Trivalvular stenosis

## Abstract

Rheumatic disease may involve the pulmonary valve, but is exceedingly rare. Simultaneous involvement of all four valves is more likely to be the result of combination of causes, such as congenital, rheumatic, infective or degenerative disease. A unitary cause for quadrivalvular involvement would be either rheumatic or myxomatous degeneration. A 16-year-old young boy presented with progressive exertional dyspnea for the past 3 years. On evaluation, he was in atrial fibrillation with congestive heart failure. On examination, evidence of stenosis of the mitral, aortic and tricuspid valves with a history of rheumatic fever in childhood was revealed. Transthoracic echocardiography showed the quadrivalvular involvement. Only few reports are available in the literature describing rheumatic quadrivalvar damage and that too in third and fourth decade. This is probably first to be reported in juvenile age group.

## Introduction

Serious stenotic lesions often occur at a single valve and multiple valves may be the site of serious disease. Rheumatic heart disease (RHD) is still the most common cause of multiple valvular lesions. Tricuspid stenosis occurs in combination with mitral stenosis (MS) in a small percentage of patients, and a few of these patients also have aortic stenosis. Patient can develop rheumatic trivalvular stenosis on a congenitally deformed pulmonic valve and may present as quadrivalvular stenosis. Rheumatic involvement of all four cardiac valves is rare [1]. Important stenosis of all four valves is rarer still; only a few case reports are available [2].

## Case Report

**Figure 1:**
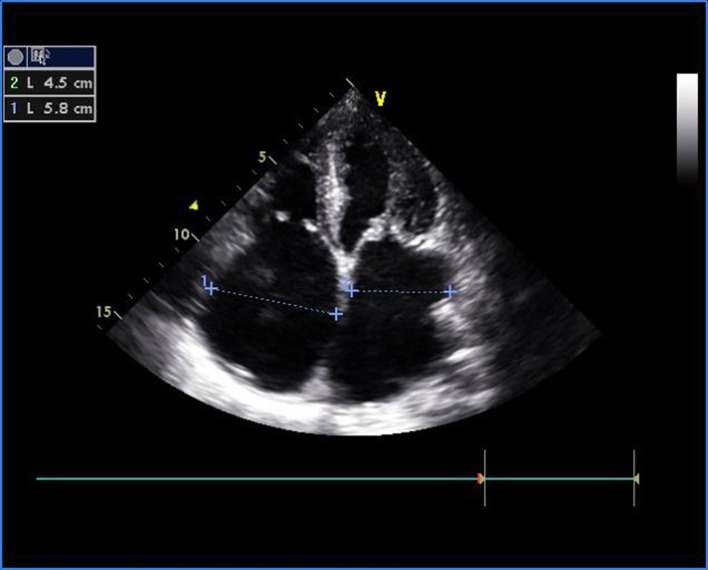
Transthoracic echocardiogram of apical four-chamber view showing thickened and doming mitral valve and tricuspid valve.

**Figure 2:**
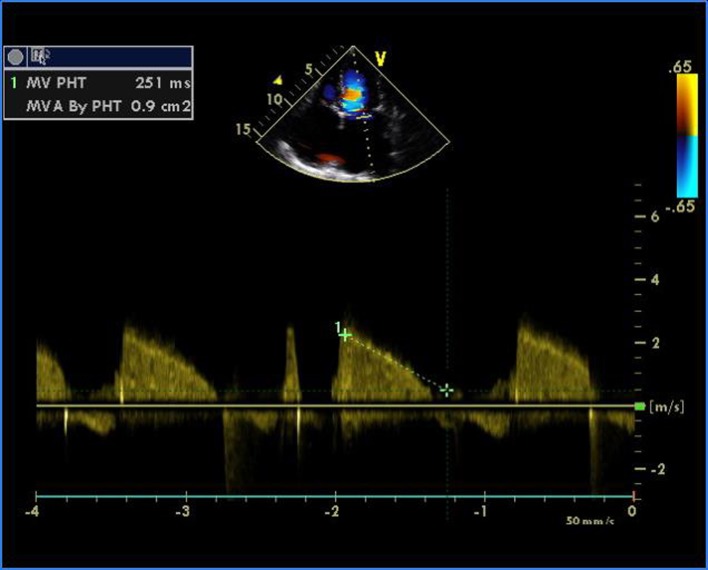
Transthoracic echocardiogram of apical four-chamber view showing severe mitral stenosis with mitral valve area of 0.9 cm^2^ by pressure half time.

**Figure 3:**
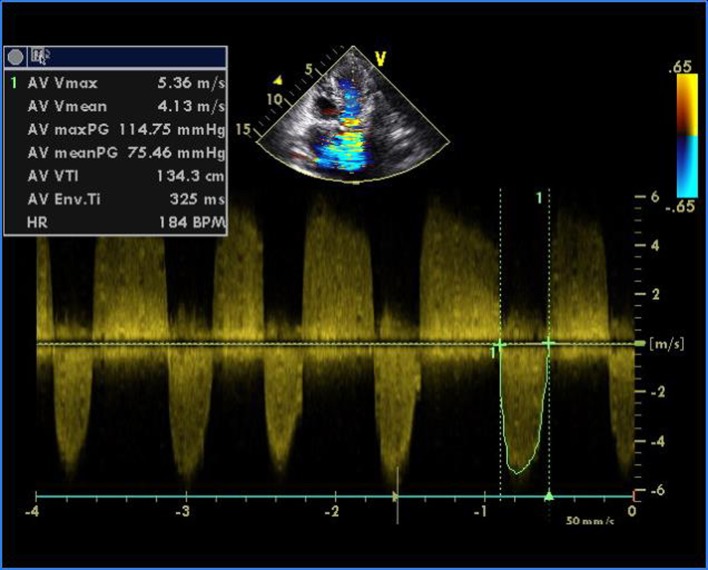
Transthoracic echocardiogram of apical five-chamber view showing severe aortic stenosis and mild regurgitation.

**Figure 4:**
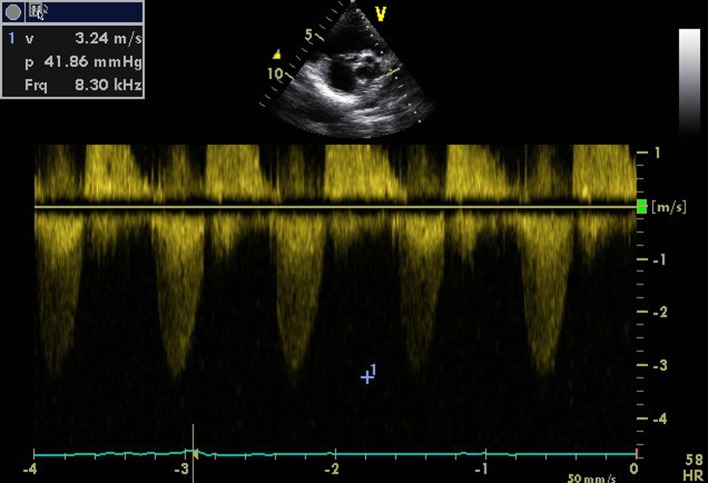
Transthoracic echocardiogram of parasternal short axis view showing pulmonic stenosis and regurgitation.

**Figure 5:**
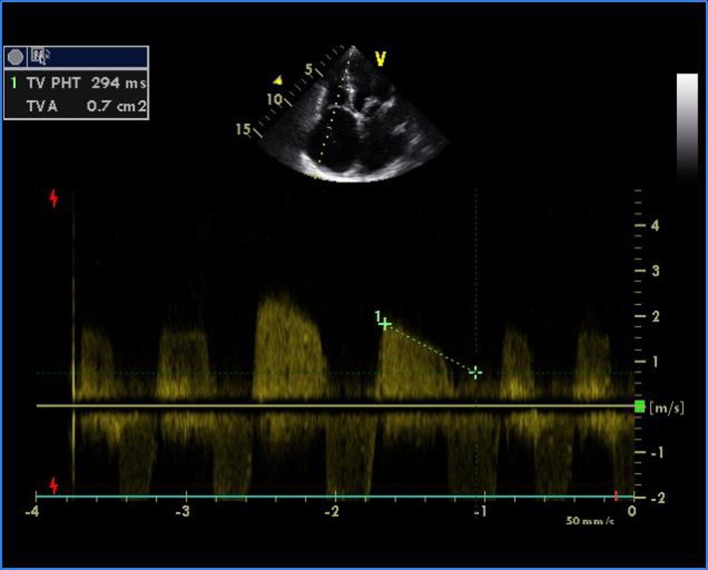
Transthoracic echocardiogram of apical four-chamber view showing severe tricuspid stenosis with valve areas of 0.7 cm^2^.

**Figure 6:**
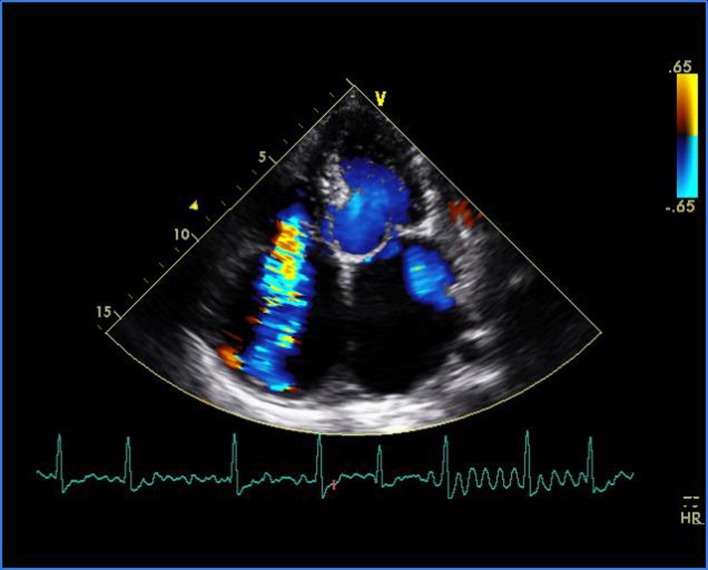
Transthoracic echocardiogram of apical four-chamber view showing severe tricuspid regurgitation and trivial mitral regurgitation.

A 16-year-old young boy presented with gradually progressive exertional dyspnea of 3 years’ duration. Initially he had New York Heart Association class I symptoms, which had gradually deteriorated to class IV at the time of presentation. He had a history of fever followed by migratory polyarthritis 9 years back. General examination revealed bilateral pedal edema. His blood pressure in right upper limb in supine posture was 102/84 mm Hg. Pulse rate was 88/min, irregularly irregular, variable in volume and normal in character. Mean jugular venous pressure was raised with absent A wave, a prominent V wave and Y descent. Apex beat was situated in fifth intercostal region, 2 cm medial to mid-clavicular line and tapping in character. The S1 was variable, and S2 was soft with split which could not be well discerned. There was an opening snap at the apex and lower left sternal border. Mid-diastolic murmurs at the apex and lower left sternal border of variable length, a grade 2 ejection systolic murmur in the aortic area conducted to the carotids, and a grade 3 pansystolic murmur at the lower left sternal border increasing on inspiration were audible. The liver was palpable 3 cm below the right costal margin with mild ascites. ECG showed right axis deviation, atrial fibrillation, and right ventricular hypertrophy. A chest radiograph in the posteroanterior view showed normal cardiac size with straightening of left border of heart, double atrial shadow with mild pulmonary venous hypertension. Transthoracic echocardiography evaluation revealed severe MS with mitral valve area of 0.9 cm^2^ and trivial mitral regurgitation. Wilkin’s score was 7/16 (M2, C1, T2, S2) (Fig. 1, 2). The mean gradient across the mitral valves was 18 mm Hg. There was severe aortic stenosis and mild regurgitation with mean systolic velocity and gradient across the aortic valve of 4.3 m/s and 75 mm Hg, respectively (Fig. 3). There was pulmonic stenosis and regurgitation with peak systolic velocity and gradient across the pulmonary valve of 3.2 m/s and 41 mm Hg respectively (Fig. 4). There was tricuspid stenosis with valve areas of 0.7 cm^2^ with mean gradient across valves of 9 mm Hg. There was severe tricuspid regurgitation (Fig. 5, 6). Biventricular function was normal with mild pulmonary artery and venous hypertension. After stabilization with drugs, he was referred to cardiothoracic surgery.

## Discussion

Acquired disease of the pulmonic valve is distinctly unusual. Any of the disease that may cause lesions of other valves rarely attacks pulmonic valve. Significant involvement of the pulmonic valve is distinctly unusual; when it occurs it is usually in patients with quadrivalvular involvement [2-5]. Involvement of all the four cardiac valves due to a rheumatic process is rare with stenosis in all valves being still more rare; only few cases have been reported [1]. Only one case of quadrivalvular disease occurred in 585 patients with RHD and only 1 in 400 patients undergoing cardiac catheterization at The New York Hospital [3, 6]. Earlier, pulmonary valve involvement was diagnosed only at surgery [5]. With advent of echocardiography, it has become much easier. Organic tricuspid valve involvement is reported to occur in more than one-third of patients with RHD studied at necropsy in patients of the Indian subcontinent [7]. There are a few reports of echocardiographic diagnosis of rheumatic cardiopathy affecting all four cardiac valves [8, 9]. Preoperative echocardiographic diagnosis of rheumatic involvement of all four cardiac valves and successful surgical treatment has also been reported [5]. Rheumatic manifestation in Indian subcontinent is different from western one notable being younger age of involvement, multivalvular affliction and fulminant course [10]. Pediatric and juvenile MS, up to the age of 12 and 20 years respectively, severe enough to require operative treatment, has been documented. These negate the belief that patients of RHD become symptomatic two decade after rheumatic fever (RF) as well as the fact that congestive cardiac failure in childhood indicates active carditis and RF. A resurgence of crippling rheumatic heart disease explains the extensive involvement of all four valves in this patient.
